# Private sector engagement in tuberculosis detection: a bibliometric analysis

**DOI:** 10.1016/j.jctube.2026.100630

**Published:** 2026-07-06

**Authors:** A.B. Duishekeeva, G.D. Dzhunushalieva

**Affiliations:** Kyrgyz State Medical Academy, 92, Akhunbaev Street, 720020 Bishkek, Kyrgyz Republic

**Keywords:** Tuberculosis, detection, private sector, public–private mix, bibliometric analysis, diagnostics

## Abstract

This study presents the first comprehensive bibliometric analysis of scientific publications in the field of the private sector's role in tuberculosis detection and diagnosis from 1964 to 2025. Using PRISMA, data were extracted from Scopus and Web of Science, yielding 616 original research articles published in 224 journals. Bibliometric performance analysis was done using Pivot Tables. Keyword co-occurrence analysis was performed using VOSviewer software to identify thematic structures. The results reveal a steady increase in publication activity since the early 2000s, reflecting growing recognition of the private sector as a critical actor in tuberculosis control. Analysis of the ten most cited articles revealed four recurring issues: suboptimal quality of care, diagnostic delays, high financial burdens on patients, and the critical need for structured public–private collaboration. The co-occurrence analysis identified five main thematic clusters. The largest cluster, “Delays and Catastrophic Costs,” highlights persistent diagnostic delays, high costs, and fragmented care within the private sector. The clusters, “Diagnostic Challenges in Private Practice” and “Case Detection and Private Practitioners”, further underscore deficiencies in diagnostic quality and coordination. Conversely, the clusters, “Public–Private Mix” and “Diagnostics and Private Providers”, demonstrate the sector's potential to expand diagnostic coverage, enhance case notification, and reduce the burden on public facilities when effective collaboration and regulatory oversight exist. Despite encouraging progress being made, we identified gaps, particularly regarding regulatory mechanisms, cost-effectiveness evaluation, and data standardisation. Strengthening governance, integrating innovative diagnostics, and incentivising quality assurance are future research directions to fully harness the private sector's contribution to ending the tuberculosis epidemic.

## Highlights

• First bibliometric study on private sector involvement in tuberculosis detection.
• Analysed 616 articles (1964–2025) from Scopus and Web of Science databases.
• Identified four thematic clusters on private sector roles in tuberculosis diagnostics.
• Found diagnostic delays and high patient costs in unregulated private care.
• Public–private mix models improve tuberculosis case detection and diagnostic coverage.

## Graphical abstract

**Figure:**
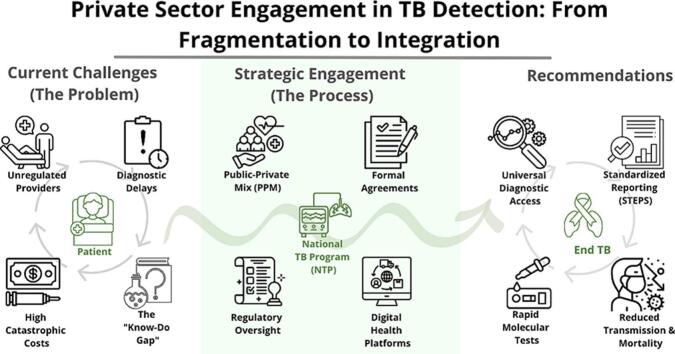

## 1 Introduction

A total of 1.25 million people died from tuberculosis (TB) in 2023, according to the WHO report [1]. There is a significant gap between the estimated number of detected TB cases and the actual number of people who are diagnosed and treated, so according to World Health Organization's (WHO) estimates, in 2023, out of 10.84 million new TB cases, 2**.7 million** went undetected by health services [2] Achieving the objectives of the End TB Strategy requires a concerted effort from society [3]. Ending the TB epidemic by 2030 is one of the health targets of the United Nations' Sustainable Development Goals (SDG 3.3) [4].

In this study, the private sector refers to all non-state healthcare providers engaged in TB diagnosis and care. Our study used a standardised WHO definition, which encompasses a broad range of participants. This included both formal entities such as private clinics, hospitals, laboratories, and pharmacies, and informal service providers such as drug sellers and unlicensed practitioners [5], [6]. Mixed organisations within the concept of “public-private partnerships” (PPPs) were also considered. This approach allows for a broad bibliometric analysis of the contribution of all service providers operating outside the direct control of the National Tuberculosis Program (NTCP), regardless of their for-profit status or official recognition.

The Public-Private Mix (PPM) strategy in tuberculosis involves integrating all healthcare providers, both public and private, into the TB control program to improve case detection and treatment outcomes [7]. It aims to ensure that individuals with TB, regardless of where they first seek care, have access to quality diagnosis and treatment [7]. TB diagnostics are a key component of global disease control [8].

For rapid TB diagnosis, molecular tests such as Xpert MTB/XDR, LPA FLD / SLD, LF-LAM, TB-LAMP assay, have been developed and approved by WHO, along with targeted next-generation (NGS) sequencing [9] and chest radiography, computer-aided detection software [10]. Involving private clinics is essential because more than half of patients in high-burden countries initially seek care in the private sector [11]. Systematic reviews show that private sector involvement improves TB detection, increases referrals to public facilities, and improves overall screening and treatment coverage [6], [11]. The roles of pharmacies and private laboratories are insufficiently integrated into mainstream research, and comprehensive evaluations of PPM implementation remain scarce outside a few high-burden countries such as Bangladesh, India, Indonesia, Kenya, Nigeria, Pakistan, and the Philippines, as noted by the WHO [12].

It is worth noting that, to date, no comprehensive bibliometric analysis has been conducted on this topic. Existing publications are mainly presented as systematic reviews, which offer a summary and critical evaluation of existing studies, but do not provide a longitudinal, quantitative view of the prevalence, dynamics, and structure of scientific connections in the field of private sector participation in the detection and diagnosis of TB [13], [14]. There are no quantitative studies in this field that cover a long period of time, demonstrate thematic clusters, and identify systemic gaps that are critical for the strategic planning of global health organisations. These gaps highlight the need for a literature review. To address these gaps, the present study examines scientific articles regarding private sector involvement in TB case detection and diagnosis (PSiTBD), following the formulated research questions:

1. What is the evolution of publications and data output on private sector involvement in TB case detection and diagnosis?
2. What contributions do the most cited publications make to the research field of private sector involvement in TB case detection and diagnosis?
3. What is the thematic structure of the research field of private sector involvement in TB case detection and diagnosis?

A bibliometric analysis is uniquely positioned to answer these research questions by mapping the intellectual structure and evolution of the PSiTBD field over several decades, including bibliometric performance analysis and keyword co-occurrence analysis. Thus, this study supplies a comprehensive perspective, giving stakeholders a strategic roadmap of where research effort has been concentrated and where urgent investigation is still required to effectively meet the End TB Strategy goals.

This article consists of four sections: an introduction, methods and materials, results that present the main findings, and a discussion with a conclusion.

## 2 Methods

**Fig. 1:**
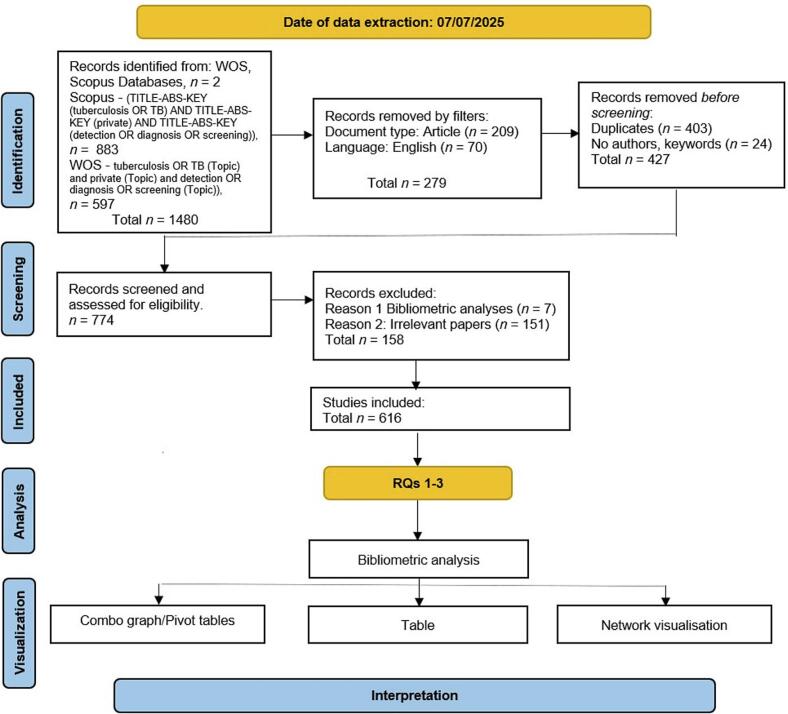
PRISMA flow diagram for selecting publications for research.

To ensure transparency and reproducibility in the process of collecting and preparing bibliometric data, we strictly followed all stages and recommendations of the PRISMA (Principles for Systematic Reviews and Meta-Analyses) [15], [16] with the plan proposed by Zupich and Čater [17] (Fig. 1). The study protocol and obtained data have been uploaded to the Open Science Framework platform (https://osf.io/eqhjm).

### 2.1 Data extraction and inclusion criteria

Bibliometric data were extracted on July 7, 2025, from Scopus and the Web of Science (WoS), two databases recognised for their comprehensive coverage and high-quality indexing of peer-reviewed scientific publications, which are also located in numerous scholarly databases, such as Medline and PubMed. These sources not only ensure the reliability of the dataset for our aims but also provide quantitative indicators for assessing the influence of articles and journals. Furthermore, both databases allow the extraction of detailed bibliometric records and enhance the depth of our study.

The search was conducted by using specific tags for titles, abstracts, and keywords. Several preliminary data extractions enabled us to select the most effective keyword combinations:

1. Scopus - *(TITLE-ABS-KEY (tuberculosis OR TB) AND TITLE-ABS-KEY (private) AND TITLE-ABS-KEY (detection OR diagnosis OR screening))*
2. WOS – (tuberculosis OR TB (Topic) and private (Topic) and detection OR diagnosis OR screening (Topic)), where Topic searches title, abstract, keyword plus, and author keywords.

The search yielded 883 bibliometric records in the Scopus database and 597 records in the WoS (Fig. 1).

### 2.2 Filtering and exclusion criteria

The filters, based on document type (Articles) and language (English), were applied to select high-quality bibliometric records best suited for bibliometric analysis. Data from two databases were merged using MS Excel tools, resulting in the identification and removal of 403 duplicates to maintain unique bibliometric records in the dataset. Records lacking keywords and authors (*n* = 24) were also excluded, as keyword co-occurrence analysis necessitated comprehensive data. To enhance reliability, independent extractions and analyses were conducted by two researchers, followed by reconciliation of findings. Discrepancies were resolved through consensus with a third reviewer.

A total of 774 articles were initially identified for screening. During this process, we excluded seven bibliometric analyses because they represented a secondary type of research. The authors conducted an independent content analysis of the article titles and abstracts, which led to the agreement to eliminate 151 irrelevant articles. Consequently, the final review included 616 articles.

### 2.3 Methods

This study utilised a three-step bibliometric approach: (1) bibliometric performance analysis (descriptive statistics) to track publication evolution and citations; (2) science mapping through keyword co-occurrence analysis to identify thematic structures; and (3) a qualitative content analysis of the top 10 most-cited articles to synthesize foundational research themes. Using the Pivot Table in MS Excel, a Combo Graph was generated that showed the evolution of publications and the top five journals. The qualitative component provides context for the quantitative findings from the full texts of the top 10 most-cited articles, which identified the main research themes and the countries/regions where research interventions occur. The co-occurrence analysis utilised VOSviewer software version 1.6.20, enabling us to create a network visualisation that helps us understand the thematic structure through colored clusters [18]. On the network visualisation, the size of each node represents the frequency of the keyword, and the links indicate the relationships between keywords [18]. Colours represent different thematic clusters.

## 3 Results

### 3.1 Overview of the data in the dataset

The findings of the descriptive analysis of the dataset highlighted the main information, as summarised in Table 1. The dataset includes a total of 616 articles published across 224 journals. The first publications in this research area in our database date back to 1964 and are available as of July 7, 2025, 585 distinct affiliations contributed to the PSiTBD research field.

**Table 1:** Main information about data.

**Overview**	#
Covered time	1964–2025
Articles	616
Journals	224
Affiliations	585
Average citations per article	20.97

**Documents Content**	
Author's keywords (DE)	2102
Keywords Plus (ID)	17,745

**Authors**	
Authors	3512
Single author	25

**Authors Collaboration**	
Co-Authors per document	5.76
International co-authors	412

The average citation count per article is 20.97, showing a decent level of impact. The analysis reveals a total of 2102 author keywords (DE) and 17,745 Keywords (Plus Tools), indicating a diverse range of research topics. The contributor pool consists of 3512 authors, but only 25 papers are credited to individual authors. On average, each paper has about 5.76 co-authors, and 412 of the articles showcase international collaboration, reflecting a strong global network in the research community.

### 3.2 The evolution of publications regarding private sector involvement in TB case detection and diagnosis

**Fig. 2:**
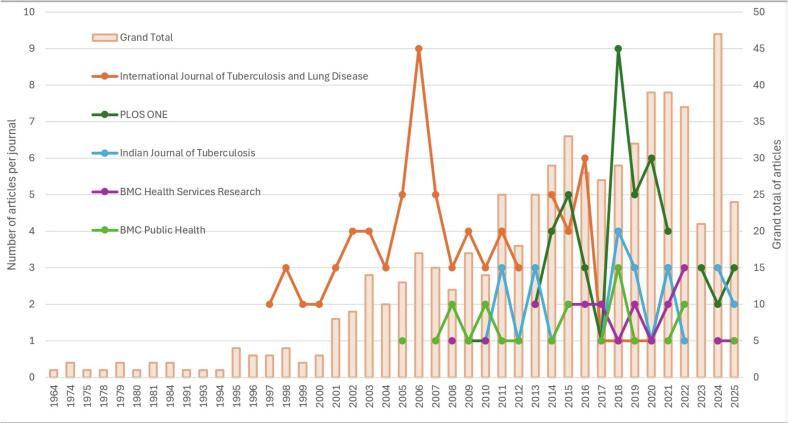
The evolution of publications (1964–2025) and the top 5 journals in the field of private sector involvement in tuberculosis detection.

The dynamics of publications by year demonstrate a steady growth of scientific interest in the PSiTBD field (Fig. 2). The light orange bars (right axis) show the annual grand total number of articles, while the colored lines (left axis) represent the number of publications per journal. Between 1964 and 2000, the number of scholarly articles remained relatively low, indicating a period of limited research activity. However, commencing in the 2000s, there has been a significant surge in publication output, culminating in a peak in 2024, which is marked by an impressive total of 48 publications per year. This upward trend suggests a growing interest and recognition of the PSiTBD field within the academic community.

The highest number of published articles on private health care organisations' role in detecting TB cases appear in the journals *International Journal of Tuberculosis and Lung Disease*, *PLOS ONE, Indian Journal of Tuberculosis*, *BMC Health Services Research,* and *BMC Public Health*, as shown in Fig. 2. The analysis identified the top five journals contributing to the PSiTBD field (32% of total), with publication counts ranging from 21 to 82 articles.

The *International Journal of Tuberculosis and Lung Disease* (82 articles) consistently contributed a substantial share of articles, particularly between 2000 and 2010, reaching a peak of 9 articles in 2006. Since 2010, PLOS ONE has emerged as a significant venue (49 articles), with a sharp increase in publications around 2017–2019. The Indian Journal of Tuberculosis shows a stable but moderate contribution (27 articles), particularly after 2010. Similarly, BMC Public Health (21 articles) and BMC Health Services Research (21 articles) have provided outlets for TB-related work focusing on health systems, services, and public health perspectives.

### 3.3 Qualitative analysis of the 10 most cited articles

The qualitative analysis of the 10 most cited articles (Table 2) reveals a landscape of the private sector's role in TB control, transitioning from early clinical observations of poor practice to modern strategic frameworks. Four key themes emerge from these foundational works.

**Table 2:** Overview of the 10 most cited articles.

Authors	Article title	Journal	Country/Region	Main research theme(s)	Year	Total citations
Broekmans JF; Migliori GB; Rieder HL; Lees J; Ruutu P; Loddenkemper R; Raviglione MC	European framework for tuberculosis control and elimination in countries with a low incidence	European Respiratory Journal	Europe	Policy/strategy; private sector commitment	2002	306
Raviglione, MC	The TB epidemic from 1992 to 2002	Tuberculosis	Global	Global Epidemiology; Policy Constraints; Exclusion of Private Sector	2003	219
Rajeswari R; Chandrasekaran V; Suhadev M; Sivasubramaniam S; Sudha G; Renu G	Factors associated with patient and health system delays in the diagnosis of tuberculosis in south india	International Journal of Tuberculosis and Lung Disease	India (South India)	Diagnostic delays (health system & patient); provider type	2002	184
Das J; Kwan A; Daniels B; Satyanarayana S; Subbaraman R; Bergkvist S; Das RK; Das V; Pai M	Use of standardised patients to assess quality of tuberculosis care: a pilot, cross-sectional study	Lancet Infectious Diseases	India (Delhi)	Quality of care assessment; know-do gap; standardised patients	2015	183
Rajeswari R; Balasubramanian R; Muniyandi M; Geetharamani S; Thresa X; Venkatesan P	Socio-economic impact of tuberculosis on patients and family in india	International Journal of Tuberculosis and Lung Disease	India	Socio-economic impact; cost of private care	1999	178
MacPherson P; Houben RMGJ; Glynn JR; Corbett EL; Kranzer K	Pre-treatment loss to follow-up in tuberculosis patients in low- and lower-middle-income countries and high-burden countries: a systematic review and meta-analysis	Bulletin of The World Health Organization	Multi-country (Africa, Asia)	Pre-treatment Loss to Follow-Up (PTLFU); Patient Attrition	2014	170
Johansson E; Long NH; Diwan VK; Winkvist A.	Gender and tuberculosis control: perspectives on health seeking behavior among men and women in vietnam	Health Policy	Vietnam	Health seeking behavior; gender differentials; stigma; private services	2000	165
Wandwalo ER; M_rkve O	Delay in tuberculosis case-finding and treatment in Mwanza, Tanzania	International Journal of Tuberculosis and Lung Disease	Tanzania	Diagnostic Delays (Patient & Provider); Role of Traditional/Private Healers	2000	154
Uplekar MW; Shepard DS	Treatment of tuberculosis by private general practitioners in india	Tubercle	India (Bombay)	Private practitioner knowledge; prescribing patterns; cost	1991	154
Lawn SD; Afful B; Acheampong JW	Pulmonary tuberculosis: diagnostic delay in ghanaian adults	International Journal of Tuberculosis and Lung Disease	Ghana	Diagnostic delays (doctor delay); provider performance	1998	140

The first topic is persistent diagnostic and provider delays. Thus, several influential studies highlight that the private sector is often the first point of care, yet it is frequently associated with significant diagnostic delays [19], [20], [21], [22]. Research in Ghana [22], India [19], and Tanzania [21] demonstrates that ‘doctor delays’ or delays within private health facilities often exceed patient-side delays, primarily due to a failure to prioritise sputum microscopy or molecular testing.

The second topic is the ‘know-do gap’ and suboptimal quality of care. A critical finding across some studies [23], [24] is the disparity between provider knowledge and actual clinical performance. Despite awareness of TB protocols, private practitioners, including qualified doctors, frequently prescribe inappropriate drug regimens and under-utilise essential diagnostic tests, favouring non-specific antibiotics instead. This ‘know-do gap’ remains a central challenge for ensuring quality care outside the public sector.

The third topic is economic Impact and patient attrition. Private sector engagement is closely linked to high financial burdens and loss to follow-up. Foundational research in India indicates that costs for TB patients in private care can be up to six times higher than in public facilities [20]. Furthermore, the lack of coordination leads to pre-treatment loss to follow-up, as patients navigate fragmented care pathways without standardised reporting systems [25].

The fourth topic is the evolution of global policy and inclusion. The studies also track the strategic shift in global TB control. Early exclusion of the private sector was identified as a major programmatic obstacle [26]. Subsequent frameworks, such as the European framework for TB elimination, have since mandated structured public-private collaboration as a prerequisite for successful disease control [27].

Collectively, these studies emphasise that while the private sector is an indispensable actor in TB detection, its involvement without regulatory oversight and standardised clinical pathways leads to fragmented, costly, and delayed care.

### 3.4 Thematic structure of the field of private sector involvement in TB case detection and diagnosis

**Fig. 3:**
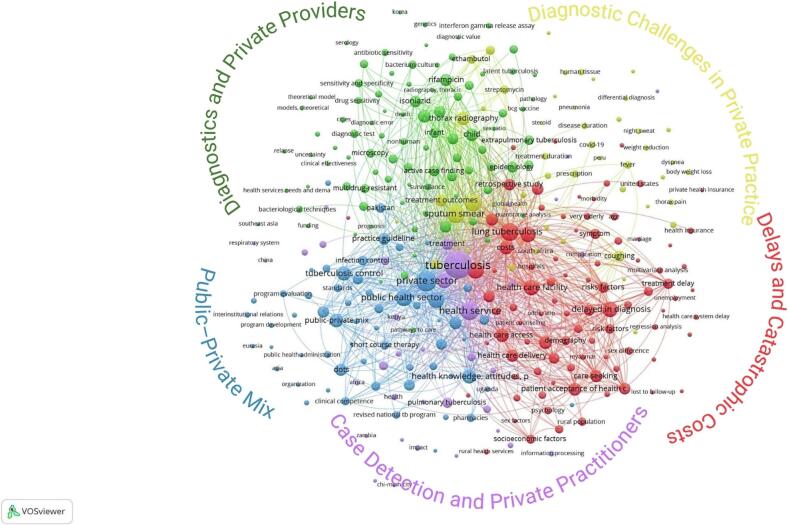
Network visualisation of thematic clusters in the field of private sector involvement in tuberculosis detection.

The keyword co-occurrence analysis revealed the main thematic areas of research focused on the private sector's participation in TB detection (Fig. 3). In the network visualisation, a total of 320 keywords is presented from an overall pool of 2848 identified keywords (with a minimum of five occurrences by default).

The red cluster is the most numerous (90 keywords) and reflects the most popular thematic area, which can be called “Delays and Catastrophic Costs”. It focuses on key themes related to “lung tuberculosis” and “risk factors” that affect “patient acceptance of health care”. It highlights significant “delays in diagnosis” and “treatment delay”, driven by both individual choices and systemic barriers. The keywords “costs” and “catastrophic costs” underscore the financial challenges faced by patients. The socio-demographic factors, including “age”, “educational status”, and “patient attitudes”, influence “care seeking” behaviours and the initiation of treatment.

The “Diagnostics and Private Providers” green cluster and “Diagnostic Challenges in Private Practice” yellow cluster focus on essential methods for TB detection, such as “sputum smear” and “microscopy”, as well as “rifampicin” and other “bacteriological techniques”. Private providers (labs and diagnostic centres) are vital in delivering timely tests and supporting drug resistance surveillance, thereby relieving the pressure on public health facilities. Keywords, “extrapulmonary tuberculosis”, “fever”, “coughing”, “weight loss”, and comorbidities such as “covid-19”, highlights the clinical complexity of TB.

The blue cluster titled “Public–Private Mix” is dominated by the keywords “private sector”, “public health sector”, and “public-private mix”, highlighting the research emphasis on collaboration between private and public systems. The cluster also features healthcare personnel, health knowledge, attitudes, practices, practice guidelines, and health programs, pointing to investigations into provider competence, adherence to national guidelines, and the implementation of standardised TB protocols, including “dots” and “short-course therapy”. Systemic dimensions are evident through terms like “organization and management”, “national health programs”, and “infection control”, which capture structural approaches to scaling up TB services.

The last smallest purple cluster, “Case Detection and Private Practitioners”, is anchored by high-frequency keywords such as “tuberculosis”, “health service”, “diagnosis”, and “treatment”, positioning it as a core thematic area on how health systems organise and deliver TB care. Within this framework, the term “private practitioner” emphasises the distinct role of independent providers, particularly in urban settings, where many patients initially seek care. The presence of case detection, cost-benefit analysis, and geographic distribution indicates research on the efficiency, equity, and effectiveness of TB detection strategies across different health service models, including the private sector.

## 4 ⁠Discussion and conclusion

This study provides a comprehensive descriptive and bibliometric analysis of publication activity concerning private sector involvement in TB case detection and diagnosis for almost 60 years. The dataset includes 616 articles published across 224 journals. Our findings align with earlier studies emphasising the growing importance of the private sector in the fight against TB, especially in resource-limited countries [28], [29].

The evolution of publications demonstrates a steady increase, particularly since the 2000s, as the private sector is increasingly recognised as a strategic partner in implementing national and global tuberculosis programs. 32% of articles have appeared in the top five productive journals, including specialised journals like the *International Journal of Tuberculosis and Lung Disease*.

The 10 most cited studies address four critical issues: the pervasive suboptimal quality of care, significant diagnostic delays, increased financial burdens on vulnerable populations, and the urgent need for strategic engagement.

Thematic structure reveals two overarching perspectives on the role of the PSiTBD, one underscoring its current limitations and the other highlighting its untapped potential. On the negative side, we observe clusters such as “Delays and Catastrophic Costs,” “Diagnostic Challenges in Private Practice,” and “Case Detection and Private Practitioners” show that private involvement is often associated with diagnostic delays, fragmented care, and high costs. Studies from Indonesia and India indicate that patients who first seek care in private facilities frequently undergo multiple visits before diagnosis and incur greater financial burdens than those accessing public services [30], [31]. Such inefficiencies are likely linked to weak regulatory oversight, inconsistent diagnostic quality, and poor coordination between private providers and national TB programmes. Limited awareness of diagnostic algorithms and financial incentives that reward treatment initiation rather than referral further exacerbate these challenges, ultimately delaying detection and increasing the risk of ongoing transmission.

Conversely, the clusters “Public–Private Mix” and “Diagnostics and Private Providers” present a more positive perspective. When effectively engaged through structured programmes, the private sector can substantially strengthen TB control by expanding diagnostic access and improving case notification. Evidence from PPM initiatives in various countries demonstrates enhanced adherence to diagnostic standards, improved reporting, and better treatment outcomes [32]. In India, for instance, the Public-Private Interface Agency model has proven both cost-effective and instrumental in scaling up access to rapid diagnostic technologies [33]. Despite the positive results of PPM-MIX in TB [34]**,** the private sector's potential remains underutilised. The publications highlight several gaps that need to be addressed.

**Geographical distribution** – few studies exist in countries with medium and low TB incidence [35], as well as in Latin American [36] and Eastern European countries [37]. Published data from rural areas are significantly lower than those from urban populations [38].

**The lack of official and systematic statistics** on the detection and individual incidence of TB in the private sector, the absence of standardised indicators for data collection, makes it difficult to monitor and observe the contribution of this part of the healthcare system to the overall incidence and treatment of TB [39], [40], [41]. Unsuccessful initiatives - there is a bias in publications, shown by the lack of reporting on unsuccessful initiatives [39], [42].

**Advanced diagnostic technologies –** the feasibility of the new Xpert MTB/XDR molecular diagnostic methods at the primary level in the private sector has not yet been demonstrated. Research is needed on how to implement best practices and disseminate the new technologies (wRDT, NGS), including sample transport systems, as well as on the implementation and testing of digital technologies for further replication and evaluation in digital interventions [43]. The knowledge-action gap – understanding why private providers know what to do but often fail to implement it (e.g., not ordering sputum tests for Xpert MTB/XDR, not performing X-rays) [44].

**Formal and informal agreements—**more research is needed on the legal status and framework of agreements between private health care providers and government entities [45]. Evaluate the advantages and disadvantages of formal arrangements versus informal agreements [45].

**Regulatory mechanisms** – how to balance strict standards with avoiding private sector exclusion [45]. Effective regulatory and subsidy systems for private providers [46].

**Incentives for private providers** – studies on effective incentives and mechanisms for notification by the private sector, including provision of drugs and consumables free of charge and financial incentives [47].

**Cost-effectiveness and impact assessment of programs** – robust, long-term studies are necessary to evaluate how different interventions (PPM, active screening, new diagnostic technologies, preventive therapy) influence TB incidence, mortality, and treatment outcomes [48]. Additionally, how various models of financing and organization of healthcare (such as integrating the private sector into government programs) affect the availability and quality of services [49], [50].

Beyond their contribution to the PSiTBD field, the findings of this bibliometric review have direct implications for clinical practice and tuberculosis treatment in the private sector. The results of the review of the most cited articles reveal a “knowledge-action gap,” suggesting that clinical education alone is insufficient. In the private sector, physicians specifically require system-wide support, such as integrated digital diagnostic algorithms and automated patient referral pathways. This approach is fundamental to ensure that knowledge is translated into the use of WHO-endorsed molecular tests and does not lead to profit-driven screening. This is demonstrated by the thematic clusters “Delays and Catastrophic Costs” and “Diagnostic Challenges,” which demonstrate a critical clinical priority: the need to move from passive case detection to more proactive, technology-driven screening in private clinics. From a clinical perspective, our results underscore that reducing diagnostic delays is far more than a programmatic goal, but a clinical necessity. This requires equipping private laboratories with rapid diagnostic methods (wRDT and NGS) and digital reporting systems. Through aligning private sector clinical protocols with standards of care that ensure a patient-centered approach (STEPS), physicians can reduce the fragmentation of care and patient loss currently documented in the literature. Thus, our bibliometric data contribute to the need to shift from unregulated, informal practices to a standardised, high-quality diagnostic environment, which is essential to achieving the goals of the End TB Strategy.

### 4.1 Limitations

It is crucial to acknowledge the limitations of our bibliometric analysis in the PSiTBD research field. First, selecting keywords poses a challenge. Although we utilised preliminary downloads to create a broad set of keywords, some relevant studies may still have been missed. Second, the specific databases we selected and our exclusion criteria could impact the comprehensiveness of our findings. We suggest that incorporating reports and conference proceedings could provide essential insights and lessons from contemporary TB programs and interventions that have been carried out.

### 4.2 Gaps and future research directions

The role of the PSiTBD faces significant challenges. There's a lack of standardised data on treatment adherence and long-term outcomes. Additionally, the informal private sector, including unregulated providers, contributes to diagnostic delays, especially in rural areas. Furthermore, special populations, like children and those with co-infections, are often overlooked, leading to gaps in necessary care and adherence to guidelines. This study highlighted critical gaps in healthcare that necessitate the development of unified interaction protocols and collaborative frameworks.

Future studies should focus on evaluating the effectiveness and cost-effectiveness of various strategies to involve the private sector, including PPM. This includes enhancing collaborative networks, standardising reporting mechanisms such as Standards of Treatment Ensuring Patient-centered care (STEPS), and incorporating new diagnostics, such as the diagnostic network optimisation. Additionally, the role of patient navigators, particularly community health workers, should be examined to understand their impact on guiding patients through complex care pathways, as well as the alignment of national insurance schemes and social support programs to reduce out-of-pocket expenses.

Behavioural interventions, utilising standardised patients, can assess how regulatory measures, such as mandatory molecular diagnostics and restrictions on antibiotic sales, influence the practices of private providers. Furthermore, it is crucial to evaluate the integration of AI-driven screening tools and their feasibility across varying patient demographics in low-resource settings, ensuring equitable access and improved health outcomes.

## CRediT authorship contribution statement

**A.B. Duishekeeva:** Writing – review & editing, Writing – original draft, Software, Investigation, Formal analysis, Data curation, Conceptualization. **G.D. Dzhunushalieva:** Writing – review & editing, Visualization, Validation, Software, Resources, Methodology, Data curation.

## Ethical statement

This study does not involve any identifiable human information or animal subjects; therefore, ethical approval and informed consent are not required. Only the publicly available body of literature was used.

## Declaration of competing interest

The authors declare that they have no known competing financial interests or personal relationships that could have appeared to influence the work reported in this paper.
